# Invasive Group B Streptococcus Disease With Recurrence and in Multiples: Towards a Better Understanding of GBS Late-Onset Sepsis

**DOI:** 10.3389/fimmu.2021.617925

**Published:** 2021-06-02

**Authors:** Mirjam Freudenhammer, Konstantinos Karampatsas, Kirsty Le Doare, Fabian Lander, Jakob Armann, Daniel Acero Moreno, Margaret Boyle, Horst Buxmann, Ruth Campbell, Victoria Chalker, Robert Cunney, Lorraine Doherty, Eleri Davies, Androulla Efstratiou, Roland Elling, Matthias Endmann, Jochen Essers, Roland Hentschel, Christine E. Jones, Steffen Kallsen, Georgia Kapatai, Marcus Krüger, Shamez Ladhani, Theresa Lamagni, Diane Lindsay, Mary Meehan, Catherine P. O’Sullivan, Darshana Patel, Arlene J. Reynolds, Claudia Roll, Sven Schulzke, Andrew Smith, Anja Stein, Axel von der Wense, Egbert Voss, Christian Wieg, Christoph Härtel, Paul T. Heath, Philipp Henneke

**Affiliations:** ^1^ Institute for Immunodeficiency, Center for Chronic Immunodeficiency, Medical Center, Faculty of Medicine, University of Freiburg, Freiburg, Germany; ^2^ Center for Pediatrics and Adolescent Medicine, Medical Center, Faculty of Medicine, University of Freiburg, Freiburg, Germany; ^3^ IMM-PACT Clinician Scientist Programme, Faculty of Medicine, University of Freiburg, Freiburg, Germany; ^4^ Paediatric Infectious Diseases Research Group, Institute of Infection and Immunity, St. George’s, University of London, London, United Kingdom; ^5^ Department of Pediatrics, University Hospital and Medical Faculty Carl Gustav Carus, Technische Universität (TU) Dresden, Dresden, Germany; ^6^ Department of Neonatology, Kinderkrankenhaus Amsterdamer Straße, Cologne, Germany; ^7^ Department of Health Northern Ireland, Belfast, United Kingdom; ^8^ Department of Pediatric and Adolescent Medicine, Division for Neonatology at the University Hospital Frankfurt, Frankfurt/Main, Germany; ^9^ Public Health Agency Northern Ireland, Belfast, United Kingdom; ^10^ Immunisation, Hepatitis and Blood Safety Department, Public Health England, London, United Kingdom; ^11^ Health Service Executive, Health Protection Surveillance Centre, Dublin, Ireland; ^12^ Irish Meningitis and Sepsis Reference Laboratory, Temple Street Children’s University Hospital, Dublin, Ireland; ^13^ Public Health Wales, Cardiff, United Kingdom; ^14^ National Infection Service, Public Health England, London, United Kingdom; ^15^ Department of Pediatric and Adolescent Medicine, St. Franziskus Hospital Ahlen, Ahlen, Germany; ^16^ Department of Pediatrics, University of Ulm, Ulm, Germany; ^17^ Faculty of Medicine and Institute for Life Sciences, University of Southampton and NIHR Southampton Clinical Research Facility and NIHR Southampton Biomedical Research Centre, University Hospital Southampton NHS Foundation Trust, Southampton, United Kingdom; ^18^ Department of Paediatrics and Youth Medicine, Klinikum Friedrichshafen, Friedrichshafen, Germany; ^19^ Department of Neonatology, München Klinik Harlaching and Schwabing, Munich, Germany; ^20^ Immunisation and Countermeasures Division, Public Health England, London, United Kingdom; ^21^ Scottish Microbiology Reference Laboratory, Glasgow Royal Infirmary, Glasgow, United Kingdom; ^22^ Health Protection Scotland, Glasgow, United Kingdom; ^23^ Department of Neonatology, Vest Children’s Hospital Datteln, University Witten-Herdecke, Witten-Herdecke, Germany; ^24^ Department of Neonatology, University Children’s Hospital Basel UKBB, Basel, Switzerland; ^25^ Glasgow Dental Hospital and School, University of Glasgow, Glasgow, United Kingdom; ^26^ Department of Pediatrics, University Hospital Essen, University Duisburg-Essen, Essen, Germany; ^27^ Neonatology and Pediatric Intensive Care, Altonaer Children’s Hospital, Altonaer Kinderkrankenhaus, Hamburg, Germany; ^28^ Klinik Hallerwiese-Cnopfsche Kinderklinik, Nürnberg, Germany; ^29^ Department of Neonatology, Klinikum Aschaffenburg, Aschaffenburg, Germany; ^30^ Department of Pediatrics, University of Würzburg, Würzburg, Germany; ^31^ PRIMAL (Priming Immunity at the Beginning of Life) Consortium, Freiburg/Lübeck, Germany

**Keywords:** group B Streptococcus, late-onset sepsis, microbiome, multiples, recurrence

## Abstract

Group B Streptococcus (GBS) is a common intestinal colonizer during the neonatal period, but also may cause late-onset sepsis or meningitis in up to 0.5% of otherwise healthy colonized infants after day 3 of life. Transmission routes and risk factors of this late-onset form of invasive GBS disease (iGBS) are not fully understood. Cases of iGBS with recurrence (n=25) and those occurring in parallel in twins/triplets (n=32) from the UK and Ireland (national surveillance study 2014/15) and from Germany and Switzerland (retrospective case collection) were analyzed to unravel shared (in affected multiples) or fixed (in recurrent disease) risk factors for GBS disease. The risk of iGBS among infants from multiple births was high (17%), if one infant had already developed GBS disease. The interval of onset of iGBS between siblings was 4.5 days and in recurrent cases 12.5 days. Disturbances of the individual microbiome, including persistence of infectious foci are suggested e.g. by high usage of perinatal antibiotics in mothers of affected multiples, and by the association of an increased risk of recurrence with a short term of antibiotics [aOR 4.2 (1.3-14.2), P=0.02]. Identical GBS serotypes in both recurrent infections and concurrently infected multiples might indicate a failed microbiome integration of GBS strains that are generally regarded as commensals in healthy infants. The dynamics of recurrent GBS infections or concurrent infections in multiples suggest individual patterns of exposure and fluctuations in host immunity, causing failure of natural niche occupation.

## Introduction

Group B streptococcus (GBS) is a leading cause of sepsis and meningitis in young infants worldwide (1)⁠. Globally in 2015, over 300 000 cases of invasive GBS disease (iGBS) caused 90 000 deaths in infants < 90 days of age (2)⁠ and neurodevelopmental impairment in over 10 000 children (3)⁠. IGBS is divided into early-onset sepsis (EOS), with disease onset – depending on the definition – either in the first 3 or 6 days, and late-onset sepsis (LOS) occurring thereafter and before day 90. Maternal GBS colonization can lead to EOS *via* vertical transmission at or before birth through ruptured membranes (4)⁠. Many countries have reported a reduction in the incidence of EOS after introducing administration of intrapartum intravenous antibiotics (IAP) to women at risk of transmitting GBS to their newborns (5, 6).

In contrast, the understanding of transmission and risk factors for LOS is still incomplete. Prematurity and maternal colonization increase the risk of LOS (7). In healthy infants, mother to infant transmission of GBS can continue for weeks after delivery (8). Neonatal colonization patterns matter, since colonization may precede invasive infection (9).

The host-microbiome interactions are highly dynamic in the first months of life (10). IAP has been shown to alter the composition of the infant gut microbiota (11–13). It is therefore conceivable that early distortions of the host-commensal adaptation, mediated by perinatal antibiotic exposure, may contribute to the pathogenesis of LOS. Indeed, several studies have reported an increase in GBS LOS after implementation of IAP, although this remains a controversial issue (14–16)⁠.

Two particularly instructive entities of iGBS are those occurring in infants of multiple births and those with recurrent episodes. The increased risk of multiple pregnancies for preterm delivery and adverse outcomes in general is well established (17), yet uncertainty exists about the specific association with iGBS. In part due to the lack in understanding of disease pathogenesis, there is no consensus about the management of the asymptomatic sibling of an iGBS case from a multiple birth (18)⁠. Additionally, the mechanisms underlying recurrent disease are not fully understood. Prematurity, persistent mucosal colonization and contaminated breast milk have been proposed as risk factors (19)⁠. Since the shared (multiples) or fixed (recurrence) genetic and environmental conditions may shed some light on iGBS pathogenesis, this study analyzed cases in multiples or with recurrence. From this analysis and review of published GBS cases, we derive a model for LOS pathogenesis to identify the infant at risk.

## Methods

### Study Design

*UK and Ireland:* Enhanced national surveillance of iGBS in infants under three months of age in the UK and Republic of Ireland (UKROI) was conducted between April 1, 2014, and April 30, 2015. Data were collected through the British Paediatric Surveillance Unit together with laboratories in England, Wales, Scotland, Northern Ireland and Ireland. Serotyping using latex agglutination and multilocus sequence typing (MLST) was performed by Public Health England and the Irish Meningitis and Sepsis Reference Laboratory. The study was approved by the South East Coast– Brighton and Sussex Research Ethics Committee (REC reference: 13/LO/1912; IRAS Project ID: 137959). The detailed methodology has been published (20)⁠.

*Germany and Switzerland:* Medical centers from the German Neonatal Network (GNN), consisting of 65 sites, were asked for GBS LOS cases from 2008-2020 with a recurrent course or with more than one affected multiple. This was complemented by an e-mail request to 120 additional medical centers with NICUs in Germany and Switzerland. Data were collected *via* data entry forms. Data collection was approved by the ethics committee of Freiburg University (Nr. 207/20).

### Study Definitions

*iGBS:* EOS (day 0-3), LOS (day 4-89), or very-LOS (>90 days) with GBS isolation from a normally sterile body site (blood, CSF). The narrow EOS definition was chosen, since > 90% of cases in the first week of life occur in this time frame. One episode of culture negative sepsis was included because it was highly suggestive of GBS LOS (clinical sepsis with consistent laboratory abnormalities, isolation of GBS from infant’s oropharynx, a concurrent episode of culture-positive GBS sepsis in the sibling and subsequent culture-positive relapse of iGBS).

*Index case:* The first infant among multiples with iGBS.

*Recurrent iGBS:* New episode of clinical illness in an infant associated with the isolation of GBS from a sterile site occurring after the completion of the therapy for the first occurrence.

*GBS colonization in infants:* Positive oropharyngeal, ear or rectal swab or gastric aspirate by culture or PCR.

*Interval between two recurrent iGBS episodes:* Days between completion of antibiotics and onset of subsequent iGBS.

*Duration of antibiotic treatment:* Duration of the antibiotic treatment of iGBS (penicillins or third generation cephalosporins).

*Short course of antibiotic treatment:* Treatment <10 days (21).

### Statistical Analysis

Continuous variables were presented as median and range and categorical data as numbers and percentages. Student’s t test or the Mann–Whitney U test and x2 test or Fisher’s exact test were used to compare continuous and categorical variables between groups. A univariable regression analysis was used to estimate associations with recurrent GBS infections in the UKROI cohort, after removing all the cases that died after the first GBS episode. Missing data were removed from the analysis. P value < 0.05 was considered significant. A multivariable model was produced using penalized regression to alleviate problems of accuracy associated with the small size of the database. Akaike’s Information Criteria (AIC) were used for model selection. Analyses were performed using STATA and R software.

### Literature Review of Cases With iGBS Recurrence

Medline and Embase were searched *via* Ovid from 1974 - 03/2020 for terms “Streptococcus agalactiae”, “group B strep*”, “strep* agalact*”, “GBS”, “double or recur* or episodes or relaps* or consecutive* or twice or two or three or four or five or repet*”, “Infant, Newborn/”, “newborn*”, “neonat*, “infant* adj4 (week* or day* or month* or premature or full term or postmature or preterm). Additional studies were identified in references of articles. Two cases in the current case study were previously reported and excluded from the review (22, 23).

## Results

### GBS in Infants From Multiple Births

#### UKROI Cohort

##### Demographic and Clinical Characteristics

A total of 41 iGBS cases in infants from 35 multiple birth pregnancies were identified, including six infant pairs in which both twins developed iGBS (17%) (**Table 1A**). Twelve infants from ten twin pairs had EOS, of which in two twin pairs (20%) both infants developed EOS. The median gestational age (GA) of twins with one sibling affected by GBS EOS was 35 (range 23-38) weeks. LOS was diagnosed in 29 infants from 25 twin pairs, of which in four twin pairs (16%) both infants developed iGBS. Based on a LOS incidence of 0.37 per 1000 live births in this population (20), the risk for LOS in a child from a multiple gestation with an already affected sibling was over 400 times higher. Comparison of the multiples with one versus two affected infants revealed no significant differences in GA (median 32 weeks for both groups) or birth weight (median 1620 (860-3560) g *vs.* 1685 (1070-2810) g). Median age at onset of GBS LOS was 42 (7- 86) days if one, and 27 (4- 54) days, if two infants were affected. The median interval in onset of disease between siblings was 2.5 (0- 18) days.

**Table 1A:** Clinical features of GBS infections in infants from multiple births (UKROI).

			birth						GBS LOS episode							
	Patient ID	gestational age (w)	Sex	birth weight (g)	Chorioamnio-nitis	Delivery mode	GBS maternal swab	ATX labour/birth	age at LOS onset (d)	Signs and symptoms	GBS detection	Serotype	Sequence type	ATX (d)	Management of other twin	recurrence
EOS - only one sibling with iGBS	1	35	M	2650	N	V	neg	N	0	P	B	N/A	N/A	21	N/A	
	2	23	M	610	Y	V	N/A	N	1	S	B	N/A	N/A	N/A	N/A	
	3	38	M	3425	N	V	neg	N	1	P	B	III	17	14	N/A	
	4	36	M	2030	N	V	neg	N	3	M	B/CSF	III	17	14	N/A	
	5	39	M	2700	N	V	pos	N	0	S	B	N/A	N/A	7	N/A	
	6	36	F	2110	Y	CS	neg	N/A	0	S	B	N/A	N/A	7	N/A	
	7	33	M	1875	Y	CS	neg	N	0	S	B	V	1	N/A	pA	
	8	35	F	2272	N	V	neg	N	1	S	B	N/A	N/A	7	N/A	
EOS - all siblings with iGBS	9a	36	M	N/A	N	V	neg	N	1	S	B	III	NA	14	SM	
	9b	36	F	N/A	N	V	neg	N	1	M	B/CSF	III	17	21	SM	
	10a	33	F	1760	N	CS	neg	Y	0	S	B	N/A	N/A	7	SM	
	10b	33	M	1870	N	CS	neg	Y	0	M	B/CSF	N/A	N/A	14	SM	
LOS - only one sibling with iGBS	11	26	M	1060	N/A	N/A	N/A	N/A	80	S	B	III	19	10	CE	
	12	37	M	3140	N/A	N/A	N/A	N/A	53	S	B	N/A	N/A	10	CE	
	13	34	M	2040	N/A	N/A	N/A	N/A	42	S	B	III	19	N/A	CE	
	14	30	F	1440	N/A	N/A	N/A	N/A	85	S	B	N/A	N/A	N/A	N/A	
	15	32	M	1550	N/A	N/A	N/A	N/A	72	S	B	N/A	N/A	14	N/A	
	16	26	M	940	N/A	N/A	N/A	N/A	49	S	B	VI	17	N/A	N/A	
	17	34	F	2150	N/A	N/A	N/A	N/A	31	M	B/CSF	III	17	21	N/A	
	18	34	F	1690	N/A	N/A	N/A	N/A	35	S	B	N/A	N/A	28	N/A	
	19	36	F	2305	N/A	N/A	N/A	N/A	7	M	B/CSF	Ia	23	14	N/A	
	20	32	M	1150	N/A	N/A	N/A	N/A	21	S	B	N/A	N/A	14	pA	
	21	37	F	2780	N/A	N/A	N/A	N/A	52	M	B/CSF	III	17	21	CE	
	22	36	F	1785	N/A	N/A	N/A	N/A	86	S	B	N/A	N/A	14	N/A	
	23	25	M	860	N/A	N/A	N/A	N/A	46	S	B	N/A	N/A	15	N/A	
	24	38	F	3560	N/A	N/A	N/A	N/A	70	S	B	N/A	N/A	32	N/A	
	25	28	F	1115	N/A	N/A	N/A	N/A	20	S	B	III	17	14	N/A	
	26	32	M	1940	N/A	N/A	N/A	N/A	42	S	B	Ia	23	7	N/A	
	27	35	F	N/A	N/A	N/A	N/A	N/A	35	S	B	III	17	21	CE	
	28	34	M	2360	N/A	N/A	N/A	N/A	32	M	B/CSF	III	17	15	pA	
	29	27	F	890	N/A	N/A	N/A	N/A	66	M	B/CSF	Ia	23	14	N/A	
	30	30	F	1450	N/A	N/A	N/A	N/A	17	S	B	Ia	23	8	CE	
	31	29	F	1095	N/A	N/A	N/A	N/A	25	M	B/CSF	N/A	N/A	21	pA	
LOS - all siblings with iGBS	32a	38	F	2620	N	CS	neg	N	5	S	B	III	17	10	NpA	
	32b	38	M	2810	N	CS	neg	N	4	M	B/CSF	III	17	14	NpA	
	33a	32	M	1750	N/A	N/A	N/A	N/A	31	M	B/CSF	III	17	14	N/A	
	33b	32	M	2005	N/A	N/A	N/A	N/A	13	S	B	III	17	14	N/A	
	34a	30	M	1620	N/A	N/A	N/A	N/A	50	S	B	III	17	21	N/A	
	34b	30	M	1070	N/A	N/A	N/A	N/A	54	M	B/CSF	III	17	21	N/A	Y
	35a	33	F	1520	N/A	N/A	N/A	N/A	30	S	B	N/A	N/A	14	NpA	
	35b	33	F	1350	N/A	N/A	N/A	N/A	24	S	B	N/A	N/A	14	NpA	

ABX, antibiotics; B, Blood; CE, clinical evaluation; CS, ceasarean section; CSF, cerebrospinal fluid; S, sepsis; M, meningitis; NpA, no prophylactic ABX; P, pneumonia; pA: prophylactic ABX administered pending the results of bacterial cultures, SM Simultaneous occurrence of GBS infection; V vaginal delivery.

^1^Infants from the same family are indicated with the same number plus a,b,c.

GBS sero- and sequence-typing revealed serotype III/ST-17 in all three tested twin sets with both infants affected by LOS and in 5/12 (42%) isolates from twin cases with only one infant affected, the rest accounted for serotypes III/ST-19 (n=2), Ia/ST-23 (n=4) and VI/ST 17 (n=1). The median treatment duration was 14 (7-32) days for sepsis and 14.5 (14-21) days for meningitis.

##### Demographic and Clinical Characteristics

Twin infants had a lower birth weight (median 1828 *vs* 3230 g, P < 0.001) were more often born prematurely (85% *vs* 24%, P < 0.001), and developed iGBS significantly later (median 25 days vs 1 day, P < 0.001) than singletons (**Table S1**). The relapse rate was similar (2.4% *vs* 1.7%, P = 0.5).

##### Demographic and Clinical Characteristics

Data on the management of the asymptomatic twin sibling of an index case were available for 12 twin pairs. Eight infants were clinically evaluated and antibiotics were not started, two of these developed iGBS. In four cases antibiotic treatment was preemptively administered to the second twin and stopped after confirmation of negative cultures.

#### German/Swiss Cohort

##### Demographic and Clinical Characteristics

Seven sets of twins and two sets of triplets, a total of 20 infants, with GBS LOS were identified (**Table 1B**). One twin set showed iGBS recurrence in both infants, leading to a total of 22 LOS episodes in these multiples. All infants were born prematurely, the median GA at birth was 31 (25-36) weeks. 7/20 infants (35%) were low birth weight (<2500 g, LBW) and 13/20 (65%) very low birth weight (<1500 g, VLBW). 11 (55%) infants were male. 4/9 pregnancies were di/trizygotic, 1/9 monozygotic, 2/9 were identified as dichorionic/diamniotic, for two pregnancies the data were not available. GBS cultures were negative in all mothers in whom antenatal maternal screening was performed (7/9 mothers). 7/9 mothers received perinatal antibiotics (indications: premature rupture of membranes, impending premature birth, suspected chorioamnionitis, cesarean section). The median age at onset of (first) GBS LOS was 48 (11- 118) days. The median interval of LOS onset between affected siblings was 5.5 (0-18) days. GBS grew in blood culture in 20 (91%), and in CSF-culture in five (23%) cases. Serotyping was performed in 7/20 children: serotype III was identified in four children, and serotype V in three. The isolates from twins were all of the same serotype and sequence type. Breast milk was tested in six women: three were positive for GBS by culture, one by PCR only, and two were negative. Two women were not breastfeeding when iGBS occurred in their infants. The median duration of antibiotic treatment for GBS bacteremia was 14 (10-19) days and 20 (14-21) days for meningitis. 10/16 infants with detailed medical records received at least one course of antibiotics prior to onset of iGBS; four of them for more than seven days. In five children hypogammaglobulinemia was detected and normalization was documented in two children. Recurrence of GBS LOS occurred in four children (25%).

**Table 1B:** Clinical features of GBS infections in infants from multiple births (German/Swiss cohort).

		Birth										GBS LOS episode												
	Patient ID^1^	gestational age (w)	zygocity/chorionicity	Sex	birth weight (g)	Chorioamnionitis	Delivery mode	GBS maternal swab	ATX labour/birth	indication for ATX	ATX before LOS (d)	age at LOS onset (d)	onset at HM/HS	Signs and symptoms	GBS detection	breastfeeding/GBS in breast milk	infant skin/mucosal swabs	Serotype	Sequence type	ATX (d)	outcome	ATB mother	continuation of breastfeeding	recurrence
LOS - all children affected	36a	31	N/A	F	1820	N	CS	N	Y	Sc	4	28	HS	S	B	Y/NA	neg	N/A	N/A	10	G	N	N	N
	36b	31	N/A	F	1440	N	CS	N	Y	SC	0	16	HS	M	B/CSF	Y/NA	neg	N/A	N/A	16	NS	N	N	N
	37a	26	DZ	M	890	Y	CS	N/A	Y	PR	2	84	HM	AA	B	N/-	N/A	N/A	N/A	N/A	L/C	N	F	N
	37b	26	DZ	F	810	Y	CS	N/A	Y	PR	2	84	HM	S	–	N/-	pos	N/A	N/A	5	N/A	N	F	Y
	38a	28	DZ	M	1125	N	CS	N	Y	PR	3	48	HS	Cl	B/CSF	Y/neg	neg	III	17	19	G	N	Y	Y
	38b	28	DZ	M	1190	N	CS	N	Y	PR	3	48	HS	Cl	B	Y/neg	neg	III	17	19	G	N	Y	N
	39a	26	DZ	M	800	Y	CS	N	Y	PR	24	117	HM	S	B	Y/neg	neg	V	N/A	14	G	R	N	N
	39b	26	DZ	M	850	Y	CS	N	Y	PR	8	112	HM	M	B/CSF	Y/neg	pos	V	N/A	21	G	R	N	N
	39c	26	DZ	M	890	Y	CS	N	Y	PR	20	118	HM	S	B	Y/neg	neg	V	N/A	14	G	R	N	N
	40a	32	DC	F	1730	N	CS	N	Y	PR	0	35	HS	S	B	Y/pos	N/A	N/A	N/A	14	G	Y	N	Y
												64	HM	S	B	N/-	N/A	N/A	N/A	14	G	N	F	–
	40b	32	DC	F	1470	N	CS	N	Y	PR	0	28	HS	M	B/CSF	Y/pos	N/A	N/A	N/A	21	G	Y	N	Y
												63	HM	M	B	N/-	N/A	N/A	N/A	14	G	N	F	–
	41a	25	DC	M	950	N	N/A	N	Y	N/A	5	14	HS	S	B	Y/pos	pos	N/A	N/A	14	G	N	Y *	N
	41b	25	DC	M	875	N	N/A	N	Y	N/A	10	20	HS	M	B/CSF	Y/pos	pos	N/A	N/A	21	NS	N	Y *	N
	42a	32	M	F	1600	N	CS	N	N	–	0	69	HM	S	B	Y/neg	neg	N/A	N/A	16	G	N	Y	N
	42b	32	M	F	1190	N	CS	N	N	–	0	51	HM	S	B	Y/neg	neg	N/A	N/A	16	G	N	Y	N
	42c	32	M	F	1250	N	CS	N	N	–	0	58	HM	S	B	Y/neg	neg	N/A	N/A	16	G	N	Y	N
	43a	36	N/A	M	2350	N	V	N/A	N	–	N/A	11	N/A	N/A	B	N/A	N/A	III	N/A	10	G	N/A	N/A	N/A
	43b	36	N/A	M	2160	N	V	N/A	N	–	N/A	11	N/A	N/A	B	N/A	N/A	III	N/A	14	G	N/A	N/A	N/A
	44a	32	DZ	F	1600	N	CS	N	Y	Sc	N/A	N/A	N/A	N/A	B	Y/pos	N/A	N/A	N/A	N/A	G	N/A	N/A	N/A
	44b	32	DZ	M	1860	N	CS	N	Y	Sc	N/A	N/A	N/A	N/A	B	Y/pos	N/A	N/A	N/A	N/A	G	N/A	N/A	N/A

AA, acute abdomen; ATX, antibiotics; B, Blood; Cl, cellulitis; CS, caesarean section; CSF, cerebrospinal fluid; DC, dichorionic; DZ, dizygotic; G, good; HM, home; HS, hospital; L/C, laparotomy+colostoma; Me, meningitis; N/A, not available; NS, neurologic sequelae; PR, PROM; R, recommended; SP, Section Prophylaxis; S, sepsis; V, vaginal delivery *sterilization.

^1^Infants from the same family are indicated with the same number plus a,b,c.

### Recurrent GBS Disease

#### UKROI Cohort

##### Demographic and Clinical Characteristics

Twelve cases of recurrent iGBS were identified, accounting for 1.4% (12/856) of all infants with iGBS reported that year (20) (**Table 2**)⁠. Eight (67%) infants were born prematurely, and five (42%) infants were of very low birth weight (<1500 g). Ten (83%) infants were male and one (8%) infant was from a twin pregnancy. Median age at the onset of the first iGBS episode was 11 (0- 73) days. Two (17%) infants had EOS and 10 (83%) LOS as the first iGBS episode. Blood culture was positive in all cases. In two (17%) infants GBS was additionally identified in the CSF. The median duration of antibiotic therapy for the first episode was seven (5- 14) days. Recurrence of iGBS occurred at a median age of 39.5 (23- 84) days. The interval from completion of initial therapy to onset of the second episode was 13 (5-75) days. In the second episode all infants had a positive blood culture and five (42%) had also a positive CSF for GBS. The median duration of antibiotic therapy for the second episode was 21 (14-42) days. GBS capsular serotyping was performed in 17 isolates from 11 (92%) cases. Serotype III was the most commonly isolated serotype (n=7, 64%), followed by Ia, Ib and V, each identified in one (9%) infant. In eight (67%) cases isolates from both episodes were available with identical serotypes in seven out of these eight cases (88%). MLST sequencing was performed in 13 isolates from seven (58%) cases and identified ST-17 in four (57%) cases.

**Table 2:** Clinical features of cases of recurrent GBS disease.

Country	ID	Sex	BW (g)	GA (w)	DM	1st episode			2nd episode			3rd episode			Isolates		Colonisation		Breast milk		Outcome	
						age (d)	GBS	Abx	age (d)	GBS	Abx	age (d)	GBS	Abx	Sero-type	MLST	Infant	Mo-ther	GBS	Cessa-tion	Imm	Seq
Germany	1	F	565	23	CS	1	B	10	48	B	12	124	B	21	N/A	N/A	pos	pos	neg	Yes	N	no
Germany	2	F	670	25	CS	41	B	14	84	B	21	–	–	–	N/A	N/A	pos	neg	neg	No*	^	no
Germany	3	F	1230	27	V	46	B	7	62	B	10	–	–	–	N/A	N/A	neg	neg	pos	Yes**	N	no
Germany	4	F	590	23	N/A	150	B	9	166	B/CSF	42	–	–	–	N/A	N/A	neg	N/A	pos	Yes**	^^	DD
Germany	5	F	1732	32	CS	35	B	14	64	B	14	–	–	–	N/A	N/A	N/A	neg	pos	Yes	N	no
Germany	6	F	1470	32	CS	28	B/CSF	21	63	B	14	–	–	–	N/A	N/A	N/A	N/A	pos	Yes	N	no
Germany	7	F	2890	39	V	10	B	10	25	B	14	–	–	–	N/A	N/A	N/A	neg	neg	No	N/A	no
Germany	8	F	4320	37	V	1	B	15	25	B/CSF	14	–	–	–	N/A	N/A	neg	pos	N/A	No	N/A	no
Germany	9	M	3020	37	CS	1	B	14	21	B	14	–	–	–	N/A	N/A	pos	pos	N/A	No	N/A	no
Germany	10	M	3020	35	V	20	B/CSF	15	60	B	14	–	–	–	N/A	N/A	pos	neg	N/A	No	N/A	no
Germany	11	F	4190	41	V	3	B	7	24	B	14	–	–	–	N/A	N/A	N/A	neg	N/A	No	^^^	no
Germany	12	M	1125	28	CS	48	B/CSF	19	74	B/CSF	19	–	–	–	III	17	neg	pos	pos	Yes	N	no
Germany	13	N/A	1920	31	CS	47	B	10	60	B	9	–	–	–	N/A	N/A	N/A	N/A	N/A	N/A	^^	no
UKROI	14	F	1082	27	N/A	39	B	10	59	B	14	–	–	–	Ia	N/A	N/A	N/A	N/A	N/A	N/A	N/A
UKROI	15	M	3770	39	V	0	B	7	23	B	14	–	–	–	III	17	N/A	neg	N/A	N/A	N/A	N/A
UKROI	16	M	3350	42	N/A	11	B	7	25	B	40	–	–	–	III	17	N/A	N/A	N/A	N/A	N/A	N/A
UKROI	17	M	2020	31	N/A	73	B	5	84	B	42	–	–	–	N/A	N/A	N/A	N/A	N/A	N/A	N/A	N/A
UKROI	18	M	770	23	N/A	1	B	9	30	B/CSF	21	–	–	–	Ib	new	N/A	pos	N/A	N/A	N/A	D
UKROI	19	M	1070	30	V	26	B/CSF	N/A	54	B/CSF	21	–	–	–	V/III	17	N/A	N/A	N/A	N/A	N/A	N/A
UKROI	20	M	1490	29	N/A	37	B	10	59	B/CSF	21	–	–	–	III	17	N/A	N/A	N/A	N/A	N/A	N/A
UKROI	21	M	3150	40	N/A	11	B/CSF	7	38	B/CSF	21	–	–	–	III	17	N/A	N/A	N/A	N/A	N/A	N/A
UKROI	22	M	3900	41	N/A	16	B	7	28	B	14	–	–	–	III	N/A	N/A	N/A	N/A	N/A	N/A	N/A
UKROI	23	M	600	23	N/A	0	B	7	82	B	N/A	–	–	–	III	N/A	N/A	N/A	N/A	N/A	N/A	N/A
UKROI	24	M	1620	31	N/A	8	B	14	41	B	14	69	B	14	III	23	N/A	N/A	N/A	N/A	N/A	N/A
UKROI	25	F	1790	32	CS	0	B	10	23	B/CSF	21	–	–	–	V	N/A	N/A	N/A	N/A	N/A	N/A	N/A

AN, Abnormal; C, Cellulitis; CS, Ceasarean section; D, Death; DD, Developmental delay; Imm, Immunology; Me, Meningitis; N, Normal; N/A, Not available; S, Sepsis; Seq., Sequelae; V, Vaginal delivery, *Pasteurized, **Temporary (restarted after maternal decolonization), ^: lack of Memory B cells and neutropenia, ^^: transient hypogammaglobulinemia, ^^^: reduced CH50 activity (<10%).

##### Risk Factors for GBS Recurrence

A univariable logistic regression showed that VLBW [OR 6.8 (1.9-22.0), P = 0.001], preterm birth [OR 5.6 (1.7-21.4), P = 0.005], a short course of antibiotics [OR 3.2 (1.0-10.9), P = 0.04] and male sex [OR 4.4 (1.1-28.7), P = 0.05] were associated with increased risk of GBS recurrence (**Table 3**, **Table S2**). When the categorical predictor variables were modelled together in a multivariate penalized logistic regression model, the association of GBS recurrence with VLBW [OR 9.7 (2.8-33.3), P < 0.001] and a short course of antibiotics [OR 4.2 (1.3-14.2), P = 0.02] remained significant (**Tables 3** and **S2**).

**Table 3:** Univariable and Multivariable Logistic Regression analysis of recurrent GBS disease (UKROI).

	Univariable		Multivariable	
	OR (95% CI)	*P* value	OR (95% CI)	*P* value
Antibiotic course <10 d	3.2 (1.0-10.9)	0.04	4.2 (1.3-18.0)	0.02
Very Low Birth Weight (<1500 g)	6.8 (1.9-22.0)	0.001	9.7 (2.8-33.3)	<0.001
Preterm Birth (<37 weeks)	5.6 (1.7-21.4)	0.005		
Male Sex	4.4 (1.1-28.7)	0.05	3.7 (0.9-15.1)	0.06

#### German/Swiss Case Series

##### Demographic and Clinical Characteristics

Thirteen cases of recurrent iGBS were reported from ten centers in Germany and Switzerland within a 12-year period (**Table 2**). Nine (69%) infants were born prematurely (median 32, range 23- 41 weeks) and six (46%) were VLBW. Nine (69%) infants were female and five (38%) infants were from a twin pregnancy. Median age at the onset of the first iGBS was 28 (1-150) days. Three (23%) infants had EOS. GBS was isolated in the blood culture in all 13 cases. Three out of nine cases with a lumbar puncture had a concurrent meningitis. The first episode was treated with antibiotics for 14 (7-21) days. Recurrence of iGBS occurred at a median age of 44 (24-64) days. The median interval from completion of initial therapy to onset of second episode was nine (3-37) days. In three cases (25%) GBS grew in the CSF culture in the second episode in addition to the blood culture. The median duration of antibiotic therapy for the second episode was 14 (9-19) days. GBS capsular serotyping was performed in one (8%) case (serotype III ST-17). Breast milk samples from eight (62%) out of 13 tested breastfeeding women revealed a positive result (by culture or PCR) in five women during the first or second episode. In six (50%) out of 12 cases, where data were reported, breastfeeding was stopped after the first or second iGBS episode and restarted after antibiotics for GBS “eradication” in two of these six cases. In one further case breast milk was pasteurized.

Four infants were treated with antibiotics with the aim to decrease GBS colonization or prevent further bacterial infections until immunological investigations were completed. In nine infants, where immunological tests were done, two were diagnosed with transient hypogammaglobulinemia, one had reduced complement activity, and one had absent memory B cells and neutropenia.

#### Literature Review on Recurrent iGBS

##### Demographic and Clinical Characteristics

We identified 44 case reports or case series of GBS recurrence in 84 infants between 1976 and 2019 (**Table S3**). Fourteen infants (24%) were twins or triplets. 64 infants (76%) had bacteremia, 16 (19%) had meningitis with bacteremia, three (4%) only meningitis and six infants (7%) had cellulitis (5 with a positive blood culture). The median age at onset of the first episode was 15 (0-120) days. Antibiotics were administered for 10 (7-28) days for the initial episode. Of the infants, in whom information was available 42/74 (57%) were preterm, 30/50 (60%) were males, 28/42 (67%) were born vaginally.

Recurrence of GBS disease occurred at a median age of 40 (8-141) days. The median interval from completion of antibiotic therapy to onset of second episode was eight (0-54) days. 64 infants (76%) had recurrent bacteremia, 16 (19%) had meningitis with bacteremia, and three (4%) meningitis without bacteremia. Antimicrobial therapy for the second episode of iGBS was administered for 14 (10-42 days) days. A third episode of GBS disease occurred in 11 infants (13%) at a median age of 61.5 (32-120) days. Capsular serotyping was performed in 56 isolates (67%) with serotype III in 40 cases (71%). The hypervirulent clone ST-17 was found in five cases where MLST results were reported.

31 breast milk samples were tested by culture or PCR and were positive for GBS in 25 cases (81%). Mastitis was reported by eight women (32%). GBS serotypes of the 11 serotyped breast milk samples were identical to those found in the infants. In 13 (52%) cases breastfeeding was ceased. 14 mothers were treated with antibiotics for mastitis or GBS decolonization. Following treatment, the breast milk of six out of seven (86%) women did not reveal GBS (culture or PCR). 12 infants were treated with antibiotics to decrease GBS colonization. After treatment, four out of six (67%) infants had a negative GBS swab, in one case after a second course of rifampicin. Immunological investigations revealed transient hypogammaglobulinemia in three infants.

#### Comparison of the Three Cohorts With Recurrence

Recurrent iGBS cases from the UKROI, German/Swiss and literature cohort were largely similar (**Figure S1**). Fewer infants from the German/Swiss case series were male as compared to UKROI (25% *vs* 83%, P = 0.01) and the literature (25% *vs* 60%, P = 0.05). Antibiotic treatment for the first episode of iGBS was shorter in UKROI (median 7 *vs* 14 days in German/Swiss cases, P = 0.01; *vs* 12.5 days in literature, P < 0.001). Yet, antibiotic treatment for the second episode of iGBS was longer in UKROI (median 21 *vs* 14 days in German/Swiss cases, P = 0.002; *vs* 14 days in literature, P = 0.06). Meningitis in the second GBS episode was more common in UKROI compared to literature (42% *vs* 13%, P = 0.03).

### Summary of Recurrent Cases and iGBS in Multiples

Combining the UKROI and German/Swiss cohorts reveals that infants with recurrent iGBS did not differ from multiples, in which more than one sibling developed iGBS with respect to gestational age, birth weight and age of LOS onset (**Table 4**).

**Table 4:** Summary of iGBS cases with recurrence and iGBS in multiples.

	Gestational age in weeks, median (IQR)	Birth weight g, median (IQR)	Age at onset of 1^st^ iGBS in days, median (IQR)	Infants with ATX before onset of 1^st^ iGBS in %	Interval between. iGBS episodes in days, median (IQR)^a^
Multiples^b^	32 (5)	1495 (721)	33 (40)^c^	NA	4.5 (6.5)
UKROI (n=12)	33 (4)	1755 (426)	27 (25)^c^	NA	2.5 (5)
German/Swiss (n=20)	31 (6)	1220 (742.5)	48 (58)^c^	62.5^c^	5.5 (6)
**Recurrence**	31 (10)	1732 (1938)	35 (30)^c^	NA	12.5 (12)
UKROI (n=12)	31 (11)	1705 (2121)	21 (26.5)^c^	NA	13 (11)
German/Swiss (n=13)	32 (10)	1732 (1895)	40 (19)^c^	56 ^c^	9 (8)

a Multiples: Interval between siblings developing iGBS. Recurrence: Interval between completion of antibiotics for the first iGBS and onset of second iGBS for the same infant.

b Multiple sets >1 sibling affected by iGBS.

c only LOS cases.

## Discussion

Cases of iGBS in infants of multiple births and those with recurrence provide intriguing insights as to how a highly virulent bacterial species adapts, or fails to do so, to the individual mucocutaneous interface as well as to the intra-familial host-commensal community. Birth constitutes a major microbial seeding event. Subsequently, a highly dynamic network develops, where microorganisms as well as immunologically important macromolecules are exchanged between family members. This goes in parallel with neonatal immune development. Normally, GBS reaches its niche without health implications for its infant host. Up to 20% healthy infants are colonized with GBS at two months of age (8) and iGBS occurs in less than 1% of colonized infants. Recurrence rate is low [1.5% in our cohort in keeping with previous reports (24)⁠], suggesting that iGBS is usually a “singular accident” rather than the result of immune problems in handling this organism. In contrast, it might lead to acquisition of host resistance, since further GBS contacts *via* the individual flora or family members are likely to occur and in general remain without consequences.

**Figure 1:**
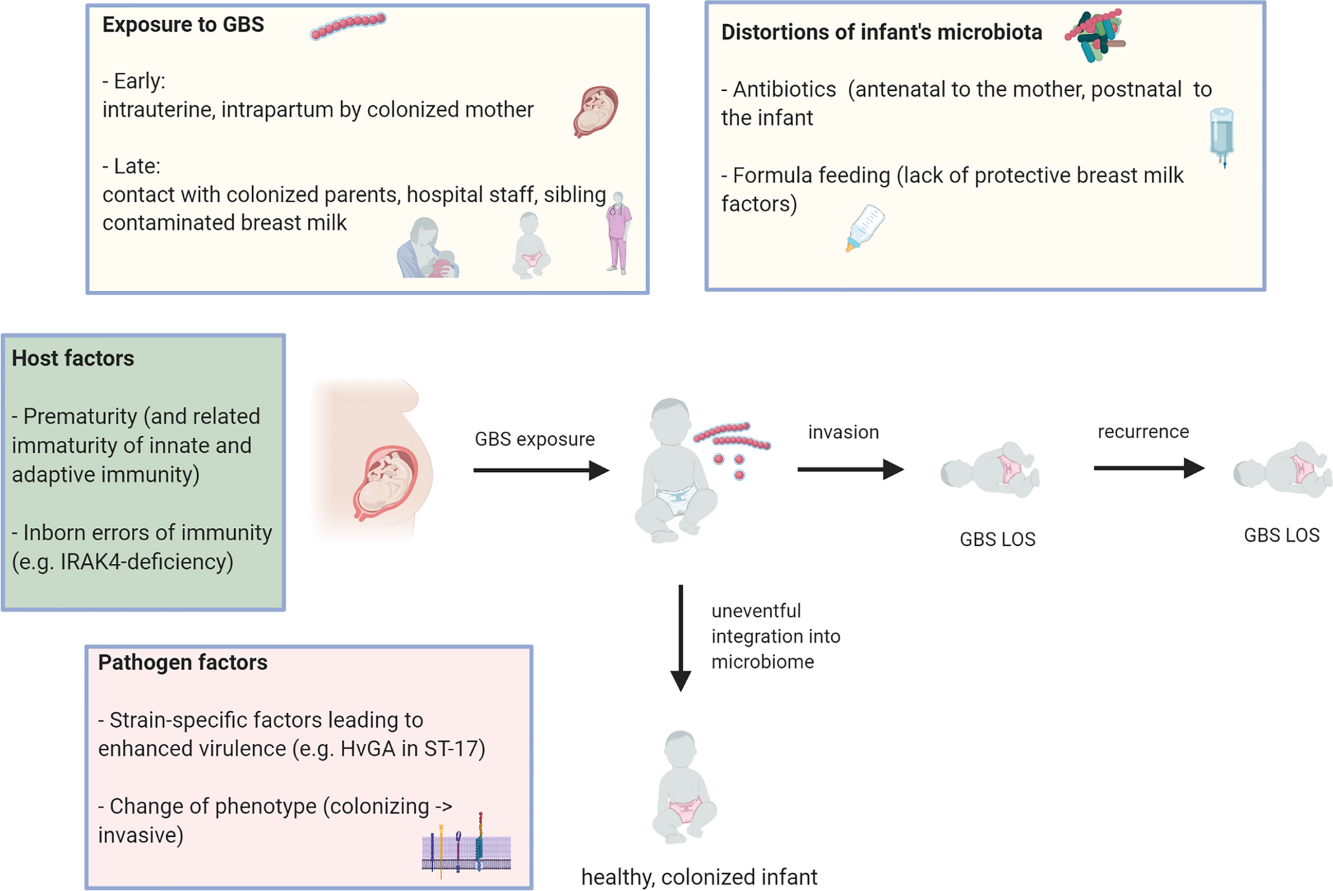
Model of pathogenesis of (recurrent) Group B streptococcus late-onset disease. Several factors (boxes) impact on the outcome of postnatal contact of the neonate with GBS: uneventful integration into microbiome *vs.* invasive GBS infection (Figure was created using BioRender.com).

Our study elucidates what might go wrong in this process. In multiples, a sibling with iGBS is a major risk factor, increasing the incidence of iGBS in the other sibling to 17%, i.e. tenfold higher as compared to the risk attributed to maternal colonization (25). The mechanisms of GBS transmission in LOS are still unclear. The main hypotheses are that acute transmission occurs after exposure to a high bacterial load (e.g. *via* breast milk) and endogenous translocation following mucosal colonization ⁠(**Figure 1**). Our data provide evidence supporting both scenarios. Simultaneous onset of iGBS in siblings within 48 hours, occurring many weeks after birth, is highly suggestive of an acute infection of both siblings from an external source, e.g. the mother. Alternatively, the infecting GBS clone may have changed its phenotype from “colonizing” to “invasive” and may be acutely transferred from one infant to the other, e.g. *via* the maternal breast. On the other hand, a long interval of up to 18 days in LOS onset between twin siblings suggests fluctuations in the individual host immunity rather than an acute infection.

Prematurity is characterized by disturbances of microbiome development, associated with frequent use of antibiotics, formula feeding and reduced contact with the maternal microbiome. It is tempting to speculate that these factors might disturb the adaptation of GBS to its neonatal host environment. Overall, maternal and subsequent neonatal GBS colonization are major risk factors for iGBS (7). Notably, in the German/Swiss multiple study, maternal antenatal rectovaginal swabs (if available) were all negative for GBS. Nearly all these women received antibiotics prior to birth, which probably reduced short term colonization with GBS, but also caused alterations of infant intestinal composition, such as a reduction in Bifidobacteria or an increase of Firmicutes species (12, 13). Accordingly, “natural” exposure to the adapted maternal GBS strain may have been missing in these infants very early in life. Moreover, antibiotics may alter bacterial virulence, which is best exemplified by the emergence of the hypervirulent GBS type III ST17 clone under tetracycline treatment pressure (26). According to a model where high GBS virulence promotes recurrence and parallel infections in multiples, ST-17 was, when molecular typing was performed, the most common clone isolated in our cohorts. The perinatal use of antibiotics may also directly disturb the development of host immunity, since antibiotics impact on neonatal myeloid cell homeostasis that provides resistance against sepsis (27). The observation that increased use of intrapartum antibiotics lowers the incidence of EOS, but may increase that of LOS is in concordance with this notion (14–16)⁠.

Invasive infections with potential pathogens like GBS with recurrence and in siblings are compatible with inherited immune aberrations. However, iGBS is usually a sporadic event and rarely uncovers genetic immunodeficiency (10, 28). Moreover, monozygosity did not stand out as a risk factor in our collection or previous reports (29). However, preterm birth is associated with various alterations of host resistance, e.g. low transplacental antibody transfer (30). Yet, immune functions do not develop in a linear fashion postnatally. For example, induced monocyte and T-cell activation is partly higher, but neutrophil function is impaired in preterm as compared to term infants and adults (31). Occasionally associated hypogammaglobulinemia, complement deficiency and neutropenia often reflect transient alterations rather than inborn errors of immunity.

Breast milk contributes to the protection against infections in various ways (32), but it is also a potential source of infection (33)⁠. GBS strains detected in breast milk were of the same serotype as the invasive strains isolated in the infants in our cohort, in case information was available. An alternative hypothesis is that mammary ducts become colonized by contact with the oral mucosa of the infant and GBS in the breast milk may just be a biomarker of the “family microbiome” (19). In general, GBS can persist at mucosal surfaces, and thus as a GBS source, even after adequate parenteral therapy (34)⁠. This concurs with the observation that the second episode is typically caused by the same serotype as the first, although this information was not available for all cases. Yet repeated translocation from the natural ecological habitat to the blood stream must be discriminated from failed eradication of truly infective foci by insufficient antibiotic treatment. Thus, the association between shorter antibiotic treatments course and increased recurrence risk, and recurrences within a week after treatment in a third of infants may highlight a subgroup of insufficiently treated cases rather than being paradigmatic for recurrence. The proportion of infants with recurrent iGBS disease that received a short course of antibiotics was disproportionately high in UKROI compared to the German/Swiss case series and the existing literature (**Figure S1**). Moreover, this finding is in disagreement with a recent study that did not show any difference in recurrence rate between shortened (≤8 days) and prolonged (>8 days) course of antibiotics among infants with uncomplicated iGBS disease. Overall, the risk of recurrence in uncomplicated iGBS is low, even if antibiotics are used for less than 8 days (35)⁠.

### How to Prevent Infection of the Asymptomatic Sibling and Recurrence in GBS Disease

Current recommendations of 10 days of intravenous (IV) antibiotics for GBS bacteremia and at least 14 days for meningitis should be adhered to (21). However, following this standard did not prevent early recurrence in several cases in our study. Moreover, and as outlined above, antibiotics in the perinatal area have undisputed costs including an increased susceptibility to sepsis. Thus, empiric antibiotic treatment in this vulnerable phase should be limited to well defined standards of antibiotic stewardship, in particular to early discontinuation if sepsis is ruled out.

The management of twins of iGBS cases has been the subject of several papers and guidelines (15, 18, 21, 36). Current NICE guidelines recommend antibiotic treatment of the other multiple(s) in case of confirmed or suspected EOS, however, no recommendations for LOS are provided (37). If a twin develops GBS EOS, the iGBS risk for the other is up to 40% (25), typically within the next 24h⁠. The short interval in GBS sepsis cases between siblings in our cohort may justify starting antibiotic treatment of the other multiple(s) in case of confirmed or suspected EOS, however, this remains a controversial issue. In any case, a careful clinical observation for the next 24 h seems reasonable (15). In contrast the interval in onset of LOS between siblings is highly variable, which makes parent education important. Routine administration of “prophylactic” antibiotics does not seem justified in LOS, given the missing evidence for a therapeutic effect and the potential costs for the host-microbiome interface (21, 36)⁠. Microbiologic investigation of breast milk and, in case of positive culture, antibiotic treatment of the mother seem appropriate in recurrent iGBS or concurrent diseases in multiples, despite lacking evidence. Additionally, probiotics may decrease the incidence of neonatal sepsis in general (38), and experimental models suggest a positive impact on GBS colonization (10, 39). Recommendations are summarized in **Table 5**.

**Table 5:** Practical recommendations for investigations and management of the asymptomatic twin in case of invasive GBS disease in a multiple birth or with recurrent course.

The recommended length of antibiotic treatment is 10 days for bacteremia without focus and 14 days for uncomplicated meningitis. A longer course is recommended when there is a complicated course.
When the sibling in a multiple birth has confirmed or suspected **invasive GBS disease**:
• using clinical judgement, consider starting antibiotic treatment of the other multiple(s) in case of confirmed or suspected EOS
• apply continuous clinical observation of the asymptomatic twin over min. 24H
• immediately start antibiotics with any sign of disease and consider stopping the antibiotics at 36 hours if the blood culture is negative, and the initial clinical suspicion of infection was not strong, and the baby’s clinical condition is reassuring and the levels and trends of biomarkers (GBC, CRP, IL-6) are reassuring
• educate the parents about the increased risk for infection in the currently asymptomatic twin and recommend prompt consultation of a doctor in case of any signs or symptoms
In case of **recurrent GBS ID** or **concurrent GBS LOS** in children of a multiple birth:
• Consider testing breast milk, discuss temporary cessation of breastfeeding or pasteurization of breast milk awaiting breast milk cultures
• If breast milk contamination is confirmed, offer eradication treatment with oral rifampicin for 5 days and retesting

## Conclusions and Outlook

The dynamics of recurrent GBS infections or concurrent infections in multiples suggest individual patterns of exposure and fluctuations in host immunity (**Figure 1**). As indicated by the low interval of iGBS in affected multiples, GBS can – probably clonally – deviate from its usually colonizing traits and become highly invasive, spreading across inter-individual boundaries. This occurs with remarkable frequency, i.e. in a sixth of multiple births in which one infant has iGBS. Identical GBS sero- and sequence types in recurrent cases and concurrently infected multiples may indicate a “streptococcal niche” at colonizing sites, which needs to be demarcated by the immune system and by competing microbes to allow for GBS to become a harmless, metabolically programmed colonizer. The necessary inter-kingdom efforts to achieve lasting coexistence are reflected by the relative long intervals between recurrent iGBS episodes in the affected (preterm) infants, who usually rapidly develop disease once invasively infected. Notably, risk factors for recurrence and simultaneous iGBS in multiples are overlapping, and iGBS in multiples seems to be a risk factor for recurrence. In order to better understand iGBS pathogenesis, it is essential to delineate the risk of empirical antibiotics, as well as the role of antibody-mediated and mucosal cellular immunity in newborn infants in unavoidable contact with GBS and other potential pathogens. It is an intriguing perspective that the improved understanding of host-microbe interface development, including the resolution of the “streptococcal niche”, will allow for the development of designer probiotics capable of improving health in the fragile neonatal period.

## Data Availability Statement

The original contributions presented in the study are included in the article/**Supplementary Material**. Further inquiries can be directed to the corresponding authors.

## Ethics Statement

The study was approved by the South East Coast– Brighton and Sussex Research Ethics Committee (REC reference: 13/LO/1912; IRAS Project ID: 137959). The detailed methodology has been published. Data collection was approved by the ethics committee of Freiburg University (Nr. 207/20).

## Author Contributions

PH, PTH, KK and MF contributed to conception and design of the study. MF and KK performed analysis of the data and wrote the first draft of the manuscript. KD helped with statistical analysis. PH, PTH, CH, and KLD revised the manuscript. MF, KK, FL, JA, DA, MB, HB, RCa, VC, RCu, LD, ED, AE, RE, ME, JE, RH, CJ, SK, GK, MK, SL, TL, DL, MM, CO’S, DP, AR, CR, SS, ASm, ASt, AW, EV and CW were involved in data acquisition. All authors contributed to the article and approved the submitted version.

## Funding

This work was supported by the Else-Kröner-Fresenius Foundation; the German Ministry of Education and Research (grants 01EO0803, 01GL1746A, 01EK1602A to PH); the German Research Council (grants HE3127/9, HE3127/12, SFB/TRR167 to PH, 413517907 as an IMM-PACT Clinician Scientist fellowship to MF) and Meningitis Now (grant 13.0189). The article processing charge was funded by the Baden-Wuerttemberg Ministry of Science, Research and Art and the University of Freiburg in the funding programme Open Access Publishing.

## Conflict of Interest

The authors declare that the research was conducted in the absence of any commercial or financial relationships that could be construed as a potential conflict of interest.
