# Supplementary material for: Invasive Group B Streptococcus Disease With Recurrence and in Multiples: Towards a Better Understanding of GBS Late-Onset Sepsis
Source: Front Immunol. 2021 Jun 2;12:617925. doi: 10.3389/fimmu.2021.617925 (PMC8208644; doi:10.3389/fimmu.2021.617925)
Supplement: Supplementary file 1 [file DataSheet_1.docx]

| **Table S1. Demographics and Clinical Characteristics of Multiples vs Singletons with iGBS in UKROI cohort** | | | |
| --- | --- | --- | --- |
|  | Twins | Singletons | *P* value |
| Cases, n | 41 | 635 |  |
| Male Sex, n (%) | 22 (54) | 338 (53) | 1 a |
| Birth weight g, median (IQR) | 1828 (925) | 3230 (1121) | <0.001 b |
| Very Low Birth Weight (<1500 g), n (%) | 12 (29) | 53 (9) | <0.001 a |
| Gestational age weeks, median (IQR) | 33 (6) | 39 (3) | <0.001 b |
| Preterm Birth (<37 weeks), n (%) | 35 (85) | 147 (24) | <0.001 a |
| Date of onset of first GBS ID days, median (IQR) | 25 (45) | 1 (16.5) | <0.001 b |
| Late onset sepsis (>72 hours) as first episode, n (%) | 30 (73) | 254 (40) | <0.001 a |
| Positive antenatal maternal swab, n (%) | 1 (8) | 33 (9) | 0.8 a |
| Chorioamnionitis, n (%) | 3 (21) | 126 (33) | 0.5 a |
| IAP, n(%) | 2 (18) | 75 (22) | 1 a |
| Meningitis, n (%) | 12 (29) | 132 (21) | 0.2 a |
| Relapses, n (%) | 1 (2.4) | 11 (1.7) | 0.5 a |
| Serotype III, n (%) | 18 (78) | 181 (60) | 0.1 a |
| Sequence Type ST-17, n (%) | 14 (61) | 140 (45) | 0.2 a |
| Duration of antibiotic treatment d, median (IQR) | 14 (6) | 10 (7) | <0.001 b |
| Antibiotic course <10 d, n (%) | 6 (17) | 192 (32) | 0.06 a |
| a Fisher's exact test.  b Mann-Whitney U test | | | |

| **Table S2. Demographics and Clinical Characteristics of Recurrence vs No Recurrence in UKROI cohort** | | | |
| --- | --- | --- | --- |
|  | Recurrence | No Recurrence | *P* value |
| Cases, n | 12 | 801 |  |
| Male Sex, n (%) | 10 (83) | 403 (53) | 0.04 a |
| Birth weight g, median (IQR) | 1705 (2121) | 3200 (1140) | 0.01 b |
| Very Low Birth Weight (<1500 g), n (%) | 5 (42) | 57 (9) | 0.004 a |
| Gestational age weeks, median (IQR) | 31 (11) | 39 (4) | 0.02 b |
| Preterm Birth (<37 weeks), n (%) | 8 (67) | 170 (26) | 0.004 a |
| Meningitis, n (%) | 2 (17) | 144 (22) | 0.7 a |
| Serotype III, n (%) | 8 (73) | 226 (61) | 0.5 a |
| Sequence Type ST-17, n (%) | 5 (71) | 177 (46) | 0.3 a |
| Duration of antibiotic treatment d, median (IQR) | 7 (3) | 10 (7) | 0.01 b |
| Antibiotic course <10 d, n (%) | 7 (58) | 191 (30) | 0.05 a |
| a Fisher's exact test.  b Mann-Whitney U test | | | |

| **Table S3 Literature Review of Recurrent GBS cases** | | | | | | | | | | | | | | | | | | | | |
| --- | --- | --- | --- | --- | --- | --- | --- | --- | --- | --- | --- | --- | --- | --- | --- | --- | --- | --- | --- | --- |
|  |  | Birth | | | | 1^st^ episode | | | 2^nd^ episode | | | 3^rd^ episode | | | GBS | Breast | |  |  |  |
| Reference | Year | Sex | GA (w) | Maternal swab | BW (g) | Age (d) | Detection of GBS | Abx (d) | Age (d) | Detection of GBS | Abx (d) | Age (d) | Detection of GBS | Abx (d) | Serotype | GBS | Cessation | Mother | Infant | Pathogenesis |
| 1 | 2019 | F | 37 | Pos (pp) | NA | 2 | B/CSF | 14 | 58 | B/CSF | 14 | NA | NA | NA | III | NA | NA | AM  (7) | AM  (14) | NA |
| 2 | 2018 | NA | 27 | Pos | 947 | 27 | B/CSF | NA | 60 | B/CSF | NA | NA | NA | NA | III | Yes (STIII) | NA | NA | NA | a |
| 3 | 2018 | NA | 40 | Neg | NA | 12 | B | 10 | 32 | B/CSF | NA | NA | NA | NA | NA | No | NA | NA | NA | NA |
|  |  | NA | 40 | Pos (pp) | NA | 0 | B | 10 | 60 | B | NA | NA | NA | NA | NA | No | NA | NA | NA | NA |
|  |  | NA | 31 | NA | NA | 23 | B | 10 | 35 | B | NA | NA | NA | NA | NA | No | NA | NA | NA | NA |
| 4 | 2018 | M | 39 | Neg | 2858 | 3 | B/CSF | 28 | 34 | B | 21 | NA | NA | NA | III | Yes (STIII) | Yes | NA | NA | b |
| 5 | 2017 | NA | T | Neg | NA | 0 | B | 11 | 16 | B | 14 | NA | NA | NA | III | No | NA | NA | NA | c |
| 6 | 2017 | F | T | Neg | NA | 8 | B | 10 | 22 | B | 10 | NA | NA | NA | Ia | Yes (STIa) | Yes | AM  (10) | RF (5) | b |
| 7 | 2017 | NA | 23 | Pos | 532 | 20 | B | 14 | 38 | B | 14 | NA | NA | NA | NA | Yes | NA | NA | NA | NA |
|  |  | NA | 24 | NA | 589 | 120 | B | 14 | 141 | B | 28 | NA | NA | NA | NA | Yes | NA | NA | NA | NA |
|  |  | NA | 30 | Neg | 925 | 25 | B | 15 | 45 | B | 28 | NA | NA | NA | Ia | NA | NA | NA | NA | NA |
|  |  | NA | 34 | Neg | 2172 | 19 | B | 14 | 37 | B | 14 | NA | NA | NA | III | Yes (STIII) | NA | NA | NA | NA |
|  |  | NA | 37 | NA | 2620 | 46 | B | 9 | 76 | B | 10 | NA | NA | NA | NA | NA | NA | NA | NA | NA |
|  |  | NA | 37 | Pos | 2530 | 64 | CSF | 16 | 109 | B | 10 | NA | NA | NA | NA | NA | NA | NA | NA | NA |
|  |  | NA | 38 | NA | 2660 | 8 | B | 7 | 22 | B | 10 | NA | NA | NA | NA | Yes | NA | NA | NA | NA |
|  |  | NA | 38 | Neg | 2914 | 9 | B | 14 | 26 | B | 21 | NA | NA | NA | NA | Yes | NA | NA | NA | NA |
|  |  | NA | 40 | Neg | 3470 | 0 | B | 10 | 43 | B | 10 | NA | NA | NA | NA | NA | NA | NA | NA | NA |
|  |  | NA | 40 | Pos | 3860 | 20 | B | 10 | 45 | B | 10 | NA | NA | NA | NA | NA | NA | NA | NA | NA |
|  |  | NA | 40 | Neg | 2814 | 25 | CSF | 17 | 96 | B | 14 | NA | NA | NA | III | No | NA | NA | NA | NA |
|  |  | NA | 41 | NA | 2840 | 21 | B | 7 | 35 | CSF | 28 | NA | NA | NA | NA | NA | NA | NA | NA | NA |
| 8 | 2016 | M | 27 | Neg | 1050 | 21 | B | 10 | 37 | B | 42 | NA | NA | NA | III | NA | NA | NA | NA | d |
| 9 | 2014 | M | 28 | Pos (pp) | 1190 | 48 | B | 19 | 74 | No | 10 | NA | NA | NA | III | Yes | Yes | NA | NA | b |
| 10 | 2013 | F | 25 | NA | 719 | 44 | B | 10 | 60 | B | 10 | NA | NA | NA | III | Yes (STIII) | Yes | AM  (10) | RF (4) | b |
| 11 | 2013 | F | 24 | NA | 640 | 54 | B | 10 | 72 | B | 14 | NA | NA | NA | IV | Yes (STIV) | Yes | AM  (10) | NA | b |
| 12 | 2013 | M | T | Pos | NA | 21 | B | 10 | 36 | B | 10 | NA | NA | NA | III | Yes (STIII) | Yes | AM  (10) | NA | b |
| 13 | 2012 | M | 29 | NA | 1465 | NA | B | 14 | NA | B | 16 | 60 | B | NA | NA | Yes | Yes | NA | NA | b,e |
| 14 | 2012 | M | 40 | Neg | NA | 2 | B | 10 | 29 | B/CSF | 21 | NA | NA | NA | III | Yes | NA | NA | NA | b |
| 15 | 2012 | M | 39 | Neg | 3112 | 43 | B | 10 | 58 | B | 14 | 74 | B | 14 | III | NA | NA | NA | NA | f |
| 16 | 2011 | NA | 27 | NA | 1140 | 25 | B | 14 | 42 | B | NA | 63 | B | NA | NA | Yes | Yes | NA | NA | b |
|  |  | NA | 26 | NA | 1066 | 40 | B | 14 | NA | B | NA | NA | B | NA | NA | No | NA | NA | NA | a |
| 17 | 2010 | F | 28 | NA | 730 | 0 | B | 14 | 38 | B | 28 | 78 | B | 21 | III | NA | NA | NA | NA | g |
| 18 | 2010 | M | 38 | Pos | NA | 0 | B | 10 | 14 | B | 14 | 32 | B | 14 | NA | Yes | P | AM  (5) | RF/C  (30) | b,f |
| 19 | 2009 | F | 31 | Pos | 1600 | 16 | B/CSF | 14 | 41 | B | 21 | NA | NA | NA | III | Yes (STIII) | P | AM  (10) | AM (21) | b |
| 20 | 2008 | F | 38 | Pos | NA | 15 | B | 10 | 30 | B/CSF | 17 | NA | NA | NA | III | Yes (STIII) | Yes | AM  (7) | NA | b |
| 21 | 2007 | F | T | NA | NA | 4 | B/CSF | 10 | 14 | B | 21 | NA | NA | NA | NA | Yes | Yes | AM  (10) | NA | f |
| 22 | 2006 | NA | 31 | NA | 1883 | 10 | B | 10 | 25 | B | 14 | NA | NA | NA | NA | Yes | Yes | AM  (7) | NA | b |
| 23 | 2005 | NA | 30 | Neg | NA | 0 | B/CSF | 21 | 30 | B | 14 | NA | NA | NA | NA | Yes | Yes | NA | NA | b |
| 24 | 2004 | M  (3/6) | NA | NA | NA | 5 (0-5)* | B | 22 (6-45)* | 30 (25-99)* | B | 24 (7-60)* | NA | NA | NA | Ia | NA | NA | NA | NA | f |
|  |  |  | NA | NA | NA |  | B |  |  | B |  | NA | NA | NA | III | NA | NA | NA | NA | f |
|  |  |  | NA | NA | NA |  | B |  |  | B |  | NA | NA | NA | III | NA | NA | NA | NA | f |
|  |  |  | NA | NA | NA |  | CSF |  |  | B |  | NA | NA | NA | III | NA | NA | NA | NA | f |
|  |  |  | NA | NA | NA |  | B |  |  | CSF |  | NA | NA | NA | III | NA | NA | NA | NA | f |
|  |  |  | NA | NA | NA |  | B/CSF |  |  | B/CSF |  | NA | NA | NA | III | NA | NA | NA | NA | f |
| 25 | 2004 | M | 26 | NA | 799 | 42 | B | 10 | 79 | B | 14 | NA | NA | NA | NA | NA | NA | NA | NA | f |
| 26 | 2003 | M | T | Pos | NA | 20 | B | 14 | #REF! | B | 21 | NA | NA | NA | NA | NA | NA | RF (4) | RF (4) | b |
| 27 | 2000 | M | 36 | Pos (pp) | 2523 | 21 | B | 10 | 35 | B | 10 | NA | NA | NA | Ia | NA | NA | NA | RF/CL (11) | f |
|  |  | M | 36 | Pos (pp) | 2296 | 20 | No | 10 | 34 | B | 10 | NA | NA | NA | Ia | NA | NA | NA | RF/CL (11) | f |
|  |  | M | 30 | NA | 1260 | 13 | B | 13 | 39 | B | 10 | NA | NA | NA | Ia | NA | NA | NA | NA | f |
|  |  | M | 38 | NA | 2836 | 26 | B | 10 | 47 | B | 10 | NA | NA | NA | III | NA | NA | NA | NA | f |
|  |  | M | 24 | NA | 800 | 59 | B/CSF | 21 | 84 | B | 21 | NA | NA | NA | III | NA | NA | NA | NA | f |
|  |  | M | 25 | NA | 490 | 112 | B | 12 | 130 | B | 12 | NA | NA | NA | V | NA | NA | NA | NA | f |
|  |  | M | 24 | NA | 783 | 24 | B | 14 | 43 | B | 21 | NA | NA | NA | V | NA | NA | NA | NA | f |
|  |  | F | 25 | NA | 680 | 25 | B | 10 | 43 | B | 14 | NA | NA | NA | V | NA | NA | NA | NA | f |
| 28 | 2000 | F | 26 | Neg | NA | 12 | B | 7 | 63 | B | 14 | NA | NA | NA | III | Yes (STIII) | Yes | AM (7) | NA | b |
| 29 | 1999 | F | 27 | NA | 959 | 35 | B | 14 | 61 | B | 11 | NA | NA | NA | Ia | NA | NA | NA | RF (4) | NA |
| 30 | 1998 | F | 27 | NA | NA | 48 | B | 14 | 75 | B | 21 | 120 | B | NA | Ia | Yes (STIa) | NA | RF  (7) | RF (7x2) | b |
| 31 | 1994 | NA | 28 | NA | 1800 | 27 | B | 13 | 48 | B/CSF | NA | NA | NA | NA | III | NA | NA | NA | NA | NA |
|  |  | NA | 34 | NA | 1460 | 1 | B | 18 | 23 | B | NA | NA | NA | NA | Ib | NA | NA | NA | NA | NA |
|  |  | NA | 30 | NA | 1130 | 1 | B | 10 | 29 | B | NA | NA | NA | NA | III | NA | NA | NA | NA | NA |
|  |  | NA | 28 | NA | 900 | 3 | B | 14 | 68 | B/CSF | NA | NA | NA | NA | III | NA | NA | NA | NA | NA |
|  |  | NA | 27 | NA | 1070 | 20 | B | 10 | 48 | B | NA | NA | NA | NA | III | NA | NA | NA | NA | NA |
|  |  | NA | 25 | NA | 700 | 15 | B | 21 | 39 | B/CSF | NA | NA | NA | NA | III | NA | NA | NA | NA | NA |
|  |  | NA | 25 | NA | 470 | 1 | B | 14 | 26 | B | NA | NA | NA | NA | Ia | NA | NA | NA | NA | NA |
|  |  | NA | 28 | NA | 1020 | 25 | B | 11 | 50 | B | NA | NA | NA | NA | III/V | NA | NA | NA | NA | NA |
|  |  | NA | 37 | NA | 2350 | 1 | B | 14 | 50 | B | NA | NA | NA | NA | Ia | NA | NA | NA | NA | NA |
| 32 | 1991 | NA | NA | NA | NA | 2 | B | 10 | 56 | B | NA | NA | NA | NA | NA | NA | NA | NA | NA | NA |
|  |  | NA | NA | NA | NA | 1 | B | 10 | 47 | B | NA | NA | NA | NA | NA | NA | NA | NA | NA | NA |
|  |  | NA | NA | NA | NA | 1 | B | 21 | 36 | B | NA | NA | NA | NA | NA | NA | NA | NA | NA | NA |
| 33 | 1989 | F | 36 | Pos (pp) | 2370 | 0 | B | 10 | 33 | B | NA | 45 | B | 15 | III | NA | NA | NA | NA | e |
| 34 | 1988 | NA | 38 | NA | 2350 | 0 | No | 7 | 26 | B | 13 | 47 | B/CSF | 18 | III | NA | NA | NA | NA | f |
| 35 | 1986 | M | 32 | Pos (pp) | 1170 | 0 | B | 18 | 62 | B | 10 | NA | NA | NA | NA | NA | NA | NA | NA | NA |
| 36 | 1985 | M | 28 | Pos (pp) | 920 | 0 | B | 10 | 42 | B | 14 | 70 | CSF | 21 | III | NA | NA | NA | RF (4) | f |
| 37 | 1982 | M | T | NA | 2552 | 30 | B/CSF | 14 | 65 | B | 21 | NA | NA | NA | III | NA | NA | NA | NA | NA |
| 38 | 1981 | M | T | NA | 3190 | 14 | B/CSF | 10 | 35 | AF | 28 | NA | NA | NA | III | NA | NA | NA | NA | h |
|  |  | M | 35 | NA | 1,925 | 3 | B | 10 | 24 | B | 12 | NA | NA | NA | III | NA | NA | NA | NA | h |
| 39 | 1979 | M | T | Pos (pp) | 2600 | 11 | B | 10 | 26 | B | 12 | NA | NA | NA | III | NA | NA | NA | NA | i |
|  |  | M | 38 | Pos (pp) | 2960 | 0 | B/CSF | 20 | 25 | B/CSF | 22 | NA | NA | NA | III | NA | NA | NA | NA | f |
|  |  | M | 32 | NA | 1350 | 47 | B/CSF | 14 | 68 | CSF | 17 | NA | NA | NA | NA | NA | NA | NA | NA | i |
| 40 | 1978 | F | 29 | Pos (pp) | 1134 | 7 | B | 14 | 35 | B/CSF | 21 | 59 | B/CSF | 21 | Ib | NA | NA | NA | NA | e |
| 41 | 1977 | M | NA | NA | 2690 | 0 | B | 10 | 49 | B/CSF | 26 | NA | NA | NA | NA | Yes | NA | OX | NA | NA |
| 42 | 1976 | M | T | Neg | 3100 | 0 | B | 10 | 20 | B/CSF | 28 | NA | NA | NA | III | NA | NA | NA | NA | g |
| 43 | 1976 | F | T | NA | 3543 | 1 | B/CSF | 8 | 8 | B/CSF | 21 | NA | NA | NA | III | NA | NA | NA | NA | i |
| 44 | 1976 | F | 37 | NA | 3200 | 8 | B/CSF | 14 | 26 | B/CSF | 31 | NA | NA | NA | NA | NA | NA | NA | NA | j |
| 45 | 1976 | F | 38 | NA | NA | 18 | B/CSF | 10 | 56 | B/CSF | 12 | NA | NA | NA | III | NA | NA | NA | NA | a |
| T: term; SN: Single; TW: Twin; TR: Triplet; pp: post=partum; B: Blood; CSF: Cerebrospinal Fluid, A: Abscess Fluid; P: Pasteurization AM: Amoxicillin, RF: Rifampicin; CL: Clindamycin; C: Cephalexin; PN: Penicillin, OX: Oxacillin NA: Not Available; *:Values expressed as median (range)  Pathogenesis: a=Horizontal transmission; b=Breast milk; c=Ingested contaminated placenta capsules containing GBS; d=Hypervirulence; e=Host immunity; f=Persistent mucosal colonization; g= Persistent infective focus; h= Penicillin tolerance; i=Underdosing; j=Short course of antibiotics | | | | | | | | | | | | | | | | | | | | |
| 1. Butler V, Pejoan H, Blot N. Recurrence of group B streptococcal meningitis. Arch Pediatr. 2019;26(6):374–6.  2. Berardi A, Guidotti I, Creti R, et al. Two Overlapping Clusters of Group B Streptococcus Late-onset Disease in a Neonatal Intensive Care Unit. Pediatr Infect Dis J. 2018;37(11):1160–4.  3. Méndez-Echevarría A, Ferreira E, Del Rosal T, et al. Difficulties in establishing the source of infection in recurrent neonatal group B streptococcal disease. Infection. 2018;46(1):141–2.  4. Ueda NK, Nakamura K, Go H, et al. Neonatal meningitis and recurrent bacteremia with group B Streptococcus transmitted by own mother’s milk: A case report and review of previous cases. Int J Infect Dis. 2018;74:13-15.  5. Buser GL, Mató S, Zhang AY, et al. Notes from the field: Late-onset infant group B streptococcus infection associated with maternal consumption of capsules containing dehydrated placenta-Oregon, 2016. MMWR Morb Mortal Wkly Rep. 2017;66(25):677–8.  6. Thomas S, Dawoud T, Doss I, et al. Recurrent late-onset group B streptococcus sepsis in a neonate from breast milk. J Clin Neonatol. 2017;6(3):192.  7. Matsubara K, Hoshina K, Kondo M, et al. Group B streptococcal disease in infants in the first year of life: a nationwide surveillance study in Japan, 2011–2015. Infection. 2017;45(4):449–58.  8. Suresh S, Tyrrell G, Alhhazmi A, et al. Recurrent sepsis and neuroinvasive disease in a neonate culture-positive for a Group B Streptococcus CPS III serotype, hvgA+ strain. JMM Case Rep. 2016;3(3):e005034.  9. Elling R, Hufnagel M, De Zoysa A, et al. Synchronous recurrence of group B streptococcal late-onset sepsis in twins. Pediatrics. 2014;133(5).  10. Davanzo R, De Cunto A, Travan L, et al. To feed or not to feed? Case presentation and best practice guidance for human milk feeding and group B streptococcus in developed countries. J Hum Lact. 2013;29(4):452–7.  11. Jawa G, Hussain Z, da Silva O. Recurrent late-onset group B Streptococcus sepsis in a preterm infant acquired by expressed breastmilk transmission: a case report. Breastfeed Med. 2013;8(1):134-136.  12. Pastore S, Zanchi C, Zanelli E, et al. Recurrent neonatal late-onset group B streptococcal disease: consider mother treatment. Pediatr Emerg Care. 2013;29(1):124.  13. Jones SM, Steele RW. Recurrent group B streptococcal bacteremia. Clin Pediatr (Phila). 2012;51(9):884-887.  14. Lombard F, Marchandin H, Jacquot A, et al. Streptococcus agalactiae late-onset neonatal infections: Should breast milk be more systematically tested for bacterial contamination? Acta Paediatr. 2012;101(12):e529-e530.  15. Shoda T, Miyagi N, Shirai K, et al. Double recurrence of group B streptococcus bacteremia in an immunocompetent infant. Pediatr Int. 2012;54(5):701–3.  16. Morinis J, Shah J, Murthy P, et al. Horizontal transmission of group B streptococcus in a neonatal intensive care unit. Paediatr Child Health (Oxford). 2011;16(6):48–50.  17. Poon WB, Lian W Bin. Recurrent group B streptococcal septicemia in a very low birth weight infant with infective endocarditis and submandibular cellulitis. Ann Acad Med Singapore. 2010;39(12):936–7.  18. Soukka H, Rantakokko-Jalava K, Vähäkuopus S, et al. Three distinct episodes of GBS septicemia in a healthy newborn during the first month of life. Eur J Pediatr. 2010;169(10):1275–7.  19. Gagneur A, Héry-Arnaud G, Croly-Labourdette S, et al. Infected breast milk associated with late-onset and recurrent group B streptococcal infection in neonatal twins: A genetic analysis. Eur J Pediatr. 2009;168(9):1155–8.  20. Gajdos V, Domelier A-S, Castel C, et al. Late-onset and recurrent neonatal Streptococcus agalactiae infection with ingestion of infected mother’s milk. Eur J Obstet Gynecol Reprod Biol. 2008;136(2):265-267.  21. Wang LY, Chen CT, Liu WH, et al. Recurrent neonatal group B streptococcal disease associated with infected breast milk. Clin Pediatr (Phila). 2007;46(6):547–9.  22. Byrne PA, Miller C, Justus K. Neonatal Group B Streptococcal Infection Related to Breast Milk. Breastfeed Med. 2006;1(4):263-270.  23. Godambe S, Shah PS, Shah V. Breast Milk as a source of Late Onset Neonatal Sepsis. Pediatr Infect Dis J. 2005;24(4):381–2.  24. Ekelund K, Konradsen HB. Invasive group B streptococcal disease in infants: A 19-year nationwide study. Serotype distribution, incidence and recurrent infection. Epidemiol Infect. 2004;132(6):1083–90.  25. Pickett KC, Gallaher KJ. Facial submandibular cellulitis associated with late-onset group B streptococcal infection. Adv Neonatal Care. 2004;4(1):20–5.  26. Kotiw M, Zhang GW, Daggard G, et al. Late-onset and recurrent neonatal Group B streptococcal disease associated with breast-milk transmission. Pediatr Dev Pathol. 2003;6(3):251–6.  27. Moylett EH, Fernandez M, Rench MA, et al. A 5-Year Review of Recurrent Group B Streptococcal Disease: Lessons from Twin Infants. Clin Infect Dis. 2000;30(2):282–7.  28. Olver WJ, Bond DW, Boswell TC, et al. Neonatal group B streptococcal disease associated with infected breast milk. Arch Dis Child Fetal Neonatal Ed. 2000;83(1):F48-F49.  29. Sabui T, Tudehope D, Lennon I. Recurrent late onset group B streptococcal infection with parotitis. J Paediatr Child Health. 1999;35(2):223–5.  30. Atkins JT, Heresi GP, Coque TM, et al. Recurrent group B streptococcal disease in infants: Who should receive rifampin? J Pediatr. 1998;132(3):537–9.  31. Green PA, Singh K V, Murray BE, et al. Recurrent group B streptococcal infections in infants: Clinical and microbiologic aspects. J Pediatr. 1994;125(6):931–8.  32. Yagupsky P, Meneges MA, Powell KR. The changing spectrum of Group B streptococcal disease in infants. Pediatr Infect Dis J. 1991;10(11):801–8.  33. Simón JL, Bosch J, Puig A, et al. Two Relapses of Group B Streptococcal Sepsis and Transient Hypogammaglobulinemia. Pediatr Infect Dis J. 1989;8(10):729–30.  34. Denning DW, Bressack M, Troup NJ, et al. Infant with two relapses of Group B streptococcal sepsis documented by DNA restriction enzyme analysis. Pediatr Infect Dis J . 1988;7(10):729–32.  35. Haque KN, Bashir O, Kambal AMM. Delayed recurrence of group B streptococcal infection in a newborn infant: A case report. Ann Trop Paediatr. 1986;6(3):219–20.  36. Millard DD, Bussey ME, Shulman ST, et al. Multiple Group B Streptococcal Infections in a Premature Infant: Eradication of Nasal Colonization With Rifampin. Arch Pediatr Adolesc Med. 1985;139(10):964.  37. Barton LL, Kapoor NK. Recurrent Group B Streptococcal Infection. Clin Pediatr (Phila). 1982;21(2):100–1.  38. Siegel JD, Shannon KM, DePasse BM. Recurrent infection associated with penicillin-tolerant group B streptococci: A report of two cases. J Pediatr. 1981;99(6):920–4.  39. Ruiz-Gomez D, Tarpay MM, Riley HD. Recurrent Group B Streptococcal Infections: Report of Three Cases. Scand J Infect Dis. 1979;11(1):35–8.  40. McCrory JH, Au-Yeung YB, Sugg VM, et al. Recurrent group B streptococcal infection in an infant: Ventriculitis complicating type Ib meningitis. J Pediatr. 1978;92(2):231-233.  41. Kenny JF, Zedd AJ. Recurrent group B streptococcal disease in an infant associated with the ingestion of infected mother’s milk. J Pediatr. 1977;91(1):158–9.  42. Broughton DD, Mitchell WG, Grossman M, et al. Recurrence of group B streptococcal infection. J Pediatr. 1976;89(2):182-185.  43. Dorand RD, Adams G. Relapse during penicillin treatment of group B streptococcal meningitis. J Pediatr. 1976;89(2):188–90.  44. Truog WE, Davis RF, George Ray C. Recurrence of group B streptococcal infection. J Pediatr. 1976;89(2):185–6.  45. Walker SH, Santos AQ, Quintero BA. Recurrence of group B III streptococcal meningitis. J Pediatr. 1976;89(2):187–8. | | | | | | | | | | | | | | | | | | | | |

**Figure S1**. Comparison of infants with recurrent iGBS from UKROI, German/Swiss cases series and literature review
