# Supplementary figures and images for: Invasive Group B Streptococcus Disease With Recurrence and in Multiples: Towards a Better Understanding of GBS Late-Onset Sepsis
Source: Front Immunol. 2021 Jun 2;12:617925. doi: 10.3389/fimmu.2021.617925 (PMC8208644; doi:10.3389/fimmu.2021.617925)

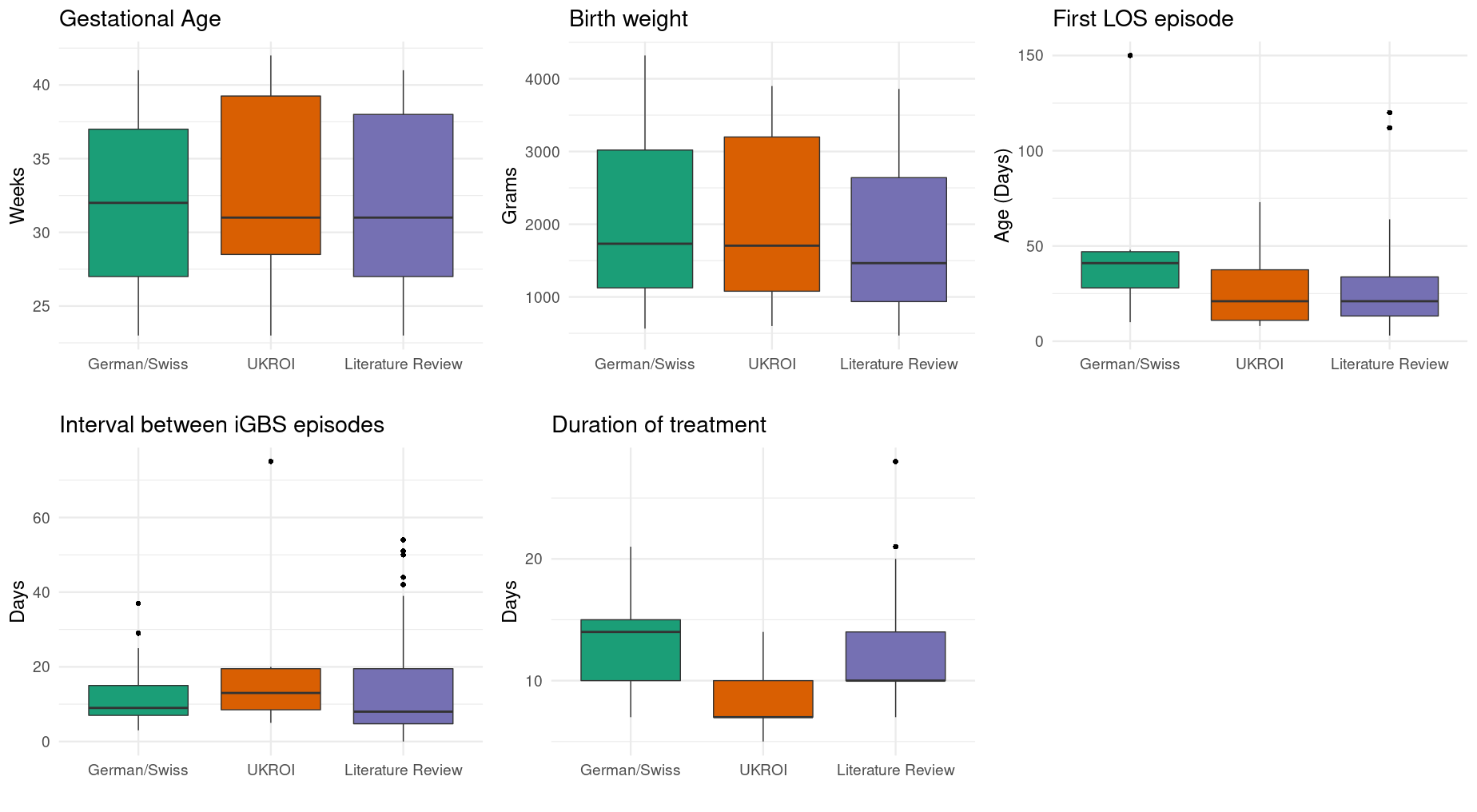

Supplement: Supplementary file 2 [file Image_1.tif]
